# Human Endometrial Organoids: Recent Research Progress and Potential Applications

**DOI:** 10.3389/fcell.2022.844623

**Published:** 2022-02-15

**Authors:** Liqun Lou, Shuangbo Kong, Yunyan Sun, Zhenbo Zhang, Haibin Wang

**Affiliations:** ^1^ Reproductive Medicine Center, Department of Obstetrics and Gynecology, Shanghai General Hospital, Shanghai Jiao Tong University, Shanghai, China; ^2^ Fujian Provincial Key Laboratory of Reproductive Health Research, Department of Obstetrics and Gynecology, The First Affiliated Hospital of Xiamen University, School of Medicine, Xiamen University, Xiamen, China; ^3^ Department of Obstetrics and Gynecology, Xinhua Hospital, Shanghai Jiao Tong University, Shanghai, China; ^4^ Shanghai Key Laboratory for Assisted Reproduction and Reproductive Genetics, Renji Hospital, Shanghai Jiao Tong University, Shanghai, China

**Keywords:** human endometrial organoids, genetic manipulation, coculture model, drug screening, epithelium

## Abstract

Since traditional two-dimensional (2D) cell culture cannot meet the demand of simulating physiological conditions *in vivo*, three-dimensional (3D) culture systems have been developed. To date, most of these systems have been applied for the culture of gastrointestinal and neural tissue. As for the female reproductive system, the culture of endometrial and oviductal tissues in Matrigel has also been performed, but there are still some problems that remain unsolved. This review highlights recent progress regarding endometrial organoids, focusing on the signal for organoid derivation and maintenance, the coculture of the epithelium and stroma, the drug screening using organoids from cancer patients, and provides a potential guideline for genome editing in endometrial organoids.

## Introduction

In recent years, many studies have focused on modeling organs *in vitro* due to the many differences between monolayer cells cultured *in vitro* and complex environments *in vivo*. Regardless of the types of constitutive cells, the polarity of the cells or the spatial structure of the tissue, the two-dimensional (2D) culture method cannot effectively simulate physiological conditions (Duval et al., 2017; Simian and Bissell, 2017). Organoid derived from human or mouse progenitor/stem cells can drive division and differentiation, and lead to the expansion of cell clones (Syed et al., 2020). These organ-specific clones will assist in the acquisition of organ/tissue precursor identity with the application of cytokines and nutrient supplements. These organoids provide a supporting structure that can sustain progenitor/stem cell self-organization as well as lineage commitment, and communication between coculture compartments can be achieved in a spatially defined manner. A large number of organoids originating from organs such as the intestine, kidney, and brain become full-grown (Lancaster and Knoblich, 2014; Koledova, 2017; Vargas-Valderrama et al., 2020); however, similar studies in regard to the female reproductive system are still in their infancy. An established endometrial organoid model replicates the characteristics of the endometrium acquiring a hollow lumen, secretion function, and apico-basal polarity (Lancaster and Knoblich, 2014), which can be used to assess normal physiological processes and perform oncology research. Resembling the endometrial epithelia, endometrial epithelial organoids comprise at least 2 cell types: ciliated and nonciliated/secretory cells; fortunately, single-cell sequencing has helped us learn more about this mixed cell population and the important signaling pathways that are vital for organoid formation (Lancaster and Knoblich, 2014; Syed et al., 2020).

## The Source of Human Endometrial Organoids

Human endometrial organoids mimic normal epithelial function and are influenced by reproductive hormones. Transcription signature expressed in organoids help us identify the cell subtypes of endometrial organoids: progenitor cells (LRIG1, PROM1, AXIN2, SOX9, SSEA-1, CADN), epithelial cells (EPCAM, KRT7, CLDN10, CDH1, LAMA4, FOXA2, KLF5, SOX17), secretory activity (PAEP, MUC1, MUC20, PAX8, KLK11, SPP1, HSD17B2), and cilia formation (FOXJ1, PIFO, RSPH1) (Valentijn et al., 2013; Nguyen et al., 2017; Turco et al., 2017; Marinic et al., 2020; Garcia-Alonso et al., 2021). In general, the endometrium models derived from normal situations are suitable for research under physiological conditions. Some studies have demonstrated that menstrual flow, which can provide tissue in a non-invasive way, contains viable cells and can successfully establish organoid with the same growing rate and transcription signature as the organoids derived from scratch biopsies (Cindrova-Davies et al., 2021). Patient-derived tumor organoids are usually used for tumor-related drug-sensitivity research because of their similar immunohistochemical and histomorphological properties. RNA-sequencing data shows the consistency of gene expression profiles between organoids derived from tumor tissues and source endometrial tumor tissue (Tamura et al., 2018). P0 cultures rather than multiply passaged cultures are recommended because P0 cultures contain almost all the cellular elements of primary tumors and no exogenous growth factors are needed (Girda et al., 2017). Endometrial stem/progenitor cells can be also derived from the decidual tissue when endometrial biopsies or elective abortion surgery is performed which initially used to optimize culture condition because they can generate high cell numbers (Turco et al., 2017). A recent study showed that organoids can be isolated from post-partum placenta tissue from patients with known pregnancy outcomes (preterm or term placenta) (Marinic et al., 2020).

During menses, removal of endometrial functionalis, residual stumps of glands of the layer basalis located at stroma regenerates intact endometrial epithelium (Ludwig and Spornitz, 1991). Evidence showed that endometrial stem or progenitor cells for epithelial regeneration anticipated in the process (Padykula, 1991). Axin2, a canonical Wnt/β catenin target gene, has been used as a marker of bipotent endometrial epithelial stem cells, and Axin2 positive epithelia form intact and functional organoids. Moreover, organoids derived from Axin2-expressing cells can be passaged steadily (Syed et al., 2020).

## Human Endometrial Organoid Culture Strategy and Pathways Involved

Human endometrial organoid basic culture medium contains advanced DMEM/F12 supplemented with serum substitute N2, B27, L-glutamate, growth factors such as fibroblast growth factor 10 (FGF10) and epidermal growth factor (EGF), antioxidants, N-acetyl-L-cysteine, Insulin-Transferrin-Selenium (ITS), PARP-1 inhibitor nicotinamide, Wnt/β-catenin signaling pathway activator and TGF-β signaling pathway inhibitor. It has been reported that the effect of nicotinamide withdrawal is more potent than the effect of the withdrawal of Noggin, R-spondin-1, A83-01, EGF and other factors (Turco et al., 2017). Wnt/β-catenin signaling pathway activation is indispensable in mammalian adult stem cell self-renewal and expansion (Yan et al., 2017). Stimulation of the Wnt/β-catenin signaling pathway is significant for endometrial epithelial stem cells to preserve stemness (Lien and Fuchs, 2014). R-spondin-1, which is a secreted protein and Wnt modulator, stimulates both Wnt/β-catenin and Wnt/noncanonical signalling. In many types of cancer, it serves as a growth promoter and plays an important role in cell proliferation, differentiation and stem cell function (Yoon and Lee, 2012). For the efficient and long-term expansion and passaging, R-spondin-1 is found to be essential with the evidence that R-spondin-1 withdrawal reduce the number and the passages of organoids (Boretto et al., 2017). In endometrial organoid culture, as an upstream regulator, R-spondin-1 can interact with the LGR4, 5 and 6, increasing phosphorylation of LRP5/LRP6 and allowing for the persistence of surface Frizzled receptors by avoiding ubiquitination, thus stabilizes β-catenin to amplifies the Wnt/β-catenin signaling pathway (Carmon et al., 2011; de Lau et al., 2014; Kessler et al., 2015; Jardé et al., 2016; Xu et al., 2020). With the shedding of the endometrium and wound healing, Wnt ligand 3 (Wnt3) expression fluctuates. The content of Wnt3 in the proliferative endometrium is nearly 4.7 times that in the secretory endometrium, which indicates that Wnt3 is engaged in progenitor/stem cell proliferation *in vivo* (Tulac et al., 2003). Indeed, both Wnt3A and R-spondin-1 are needed for organoid culture. Another WNT pathway activator added to culture medium is the glycogen synthase kinase 3 (GSK-3α/β) inhibitor CHIR99021 (Santos et al., 2019; Xin Wang et al., 2020). As an inexpensive and efficient alternative, CHIR99021 can achieve an equal effect (Haider et al., 2019). It is encouraging that some evidence has shown that Wnt3 may not be needed for the growth and expansion of endometrial organoids because of elevated and endogenously expressed Wnt ligands (Tulac et al., 2003; Punyadeera et al., 2005; Boretto et al., 2017).

It has been reported that the TGF-β signaling pathway and Wnt/β-catenin signaling pathway exhibit mutual interference (Ng-Blichfeldt et al., 2019). It has been reported that the cell cycle inhibitor p21 is a downstream gene of TGF-β signaling pathway which induces epithelial cell growth arrest in the late G1 phase and increases the apoptosis rate (Derynck, 1994; Martin et al., 2003; Niimi et al., 2007; Yoshimoto et al., 2015). The TGF-β signaling pathway induce stem cell differentiation via the activation of ALKs/Smads (Lu et al., 2017). A83-01, a transforming growth factor (TGF)-β type I receptor (TβR-I) inhibitor, strongly inhibits ALK4, ALK5 and ALK7, blocks TGF-β by reducing Smad2 phosphorylation levels thereby inhibits TGF-β induced epithelial-mesenchymal transition (Bates and Mercurio, 2003; Tojo et al., 2005; Cui et al., 2019). Noggin, a BMP-4 antagonist, binds to BMP4 tightly prevent BMP4 to interact with their receptors therefore inhibiting differentiation. Noggin is essential to maintain self-renewal characteristics in organoid culture (Kuijk et al., 2016; Urbischek et al., 2019; Phan-Everson et al., 2021). Some culture strategy proposes E2 addition can not only enhance the number of passaging but also the growth and expansion of organoids, however, it is not indispensable (Boretto et al., 2017).

## Cell Fate Determination in Human Endometrial Organoids

Human endometrial organoids are realizable and practical models to mimic the menstrual cycle. In accordance with the *in vivo* process, organoids are treated with oestrogen (E2) and medroxyprogesterone acetate (MPA). The single-cell transcriptome atlas, which depicts a high-dimensional search space, vividly illustrates different cell types in human endometrial organoids include ciliated, secretory, proliferative, stem, and unciliated cells and epithelial cells. Ciliated cells increase throughout the E2 and E2+MPA disposal which mimic artificial menstrual cycle in endometrial organoids. The number of stem cells was decreased by E2 and E2+MPA treatment, while E2+MPA increased the number of secretory cells (Fitzgerald et al., 2019). The NOTCH signaling pathway is known for cell fate determination. In a clinical study, compared with healthy fertile women, patients with endometrosis, repeated implantation failure (RIF) and polycystic ovary syndrome (PCOS), showed dysregulated NOTCH signalling expression in mid-luteal region (Amjadi et al., 2019). Other evidence in agreement with those of the clinical study showed that the NOTCH and WNT signaling pathway influence the proportion of ciliated and secretory cells. WNT targets such as FOXJ1 are highly active in the ciliated cells, however, the glandular cells show transcriptional activation induced by WNT inhibition and NOTCH activation (Garcia-Alonso et al., 2021). RNA velocities projected into t-distributed stochastic neighbor embedding (t-SNE) plots for control and Notch inhibitor dibenzazepine (DBZ; generic name iminostilbene)-treated cells revealed that DBZ treatment promotes ciliated cell differentiation and transdifferentiation from secretory cells to ciliated cells (Cochrane et al., 2020). In adult fallopian tube organoids, NOTCH signaling has been demonstrated to sustain the expression of stem cell markers. DBZ treatment increases the abundance of cilia and accessory microtubules in fallopian tubes (Kessler et al., 2015). In other human epithelial organoid models, such as urothelial organoids, Notch signaling participates in urothelial differentiation, decreasing progenitor expression (Santos et al., 2019). In an intestinal organoid model, Yap1-Notch-Dll1 axis activation drives polarization and the formation of complex multicellular asymmetric structures (Serra et al., 2019).

## Genetic Manipulation of Human Endometrial Organoids

Human endometrial organoids are embedded in Matrigel, which is similar to extracellular matrix (ECM). The ECM supports cell physiological functions but obviously hinders the efficiency of genome editing. Some regular genetic strategies, such as RNA interference (RNAi) and plasmid transfection, are widely used in mammalian cells as powerful tools to manipulate genes (Laperrousaz et al., 2018). Lipofection combined the CRISPR/Cas9 ensures that the targeting agent is incorporated into the target cells to facilitate gene knock-in or knockout. However, this traditional tool faces challenges when it is used to deal with three-dimensional (3D) models when cells are embedded in solid ECM. Some commercial reagents, such as Lipofectamine 2000, have been proven to have extremely low efficiency. Polymeric nanoparticles, which are said to be the best method, only show a transfection efficiency of 6% in 3D culture, which is a nearly 90% decrease compared with the efficiency in 2D culture (Morgan et al., 2018).

By contrast, viral vectors have a higher feasibility for use in the transfection of large plasmids or the targeting of cells that are hard to transfect through regular lipofection. However, intrinsic biosafety issues cannot always be avoided because vectors carried by lentivirus insert into the genome randomly, which may lead to gene mutation. Adenovirus transfection can circumvent this defect to some extent, and other optimization methods are emerging. Matrigel bilayer organoid culture (MBOC) is reported to achieve 90% infection efficiency (Maru et al., 2019).

Even if the success rate of virus-related genome editing is elevated, another problem still needs to be considered. The volume of Matrigel for the viral or nonviral transfection methods mentioned above should be as low as possible. The usual solution is to deprive of ECM in the formed organoid and disperse spheroids into single cells and then these cells would be reseeded into Matrigel or hydrogel again. At this point, the cells are in a 2D state, which presents another problem. Organoids derived from single precursor stem cells lose homology and polarity, which means that the gland is no longer intact (Co et al., 2021; Tsai et al., 2018). Therefore, *in situ* transfection is worthy of further study.

Compared with lipofection or lentiviral infection, electroporation for stable DNA transduction is a powerful tool to edit the genome in some human primary cells, digestive and reproductive system organoids that are hard to transfect (Fujii et al., 2015; Dekkers et al., 2021). Electroporation *in situ* in 3D matrices has been attempted in other kinds of models. Single organoids originating from glandular organs with cavity structure have been injected with an exogenous DNA mixture by capillary injection, and then tweezer electrode electroporation has been performed in human liver ductal organoids (Hendriks et al., 2021). Homology-directed repair has been successfully applied for genetic engineering using the electroporation combined with CRISPR–Cas9 (Matano et al., 2015). HDR used to be considered precise genome editing, but the efficiency is low. A recent report named CRISPR–HOT, which depends on “error-prone” nonhomologous end joining (NHEJ) for genetic engineering, achieved ten times high efficiency to make knock-in manipulation (Artegiani et al., 2020). CRISPR–Cas9 based genome engineering has allowed the knock-in and knockout of multiple genes to be achieved, and there are reasonable grounds for further study of the application of endometrial organoids (Hendriks et al., 2021).

## Coculture Models With Human Stroma

In the process of endometrial preparation and embryo implantation, the epithelium plays an important role. Although the epithelium is the first one to which embryo attaches, crosstalk between epithelial cells and stromal cells is indispensable for the proper differentiation of both cell types. The drawback for *in vitro* experience and the lack of an ideal spontaneous decidual animal model limit research on the human endometrium. Some attempts have been made to decide what kind of human endometrial stromal fibroblast cell can be cocultured with human endometrial epithelial organoids, and the key to success may be that endometrial stromal cells can stick to the spherical organoid structure and then form a compact structure. Stromal cells cocultured with epithelial organoids can be divided into several types based on the origin. It has been reported that human pluripotent stem cell-derived endometrial stromal fibroblasts (PSC-ESFs) can be cocultured with organoids in Matrigel and respond to hormone signaling transforming into decidualized endometrial stromal fibroblasts (Cheung et al., 2021). In some respect, Matrigel is not suitable ECM for fibroblast cells when co-cultured with the epithelia. Evidence showed that primary stromal cells can be cocultured with organoids in hydrogel matrix comprised 97% type I and 3% type III collagens which resembled mid-luteal endometrium in the constituent and in-use elastic modulus (Pa) of comparable magnitude (Aplin et al., 1988; Iwahashi et al., 1996; Oefner et al., 2015; Abbas et al., 2019; Rawlings et al., 2021). Recently, a new type of synthetic matrix was discovered to be more suitable for tumorous relevant organoids-ECM interactions. Synthetic hydrogel design guided by multiomic evaluation of tumor and normal tissue revealed pancreatic organoids microenvironment. Based on this, the new synthetic scaffold uses eight-arm adhesion-linker pre-functionalized vinyl sulfone-activated PEG macromer (f-PEG-vs) based hydrogel system which is sensitive to matrix metalloproteinase (MMP). The system contains the fibrillar analogue FN-mimetic peptide PHSRN-K-RGD, collagen analogue the GFOGER peptide, and a BM-binding peptide which maintains cell secreted matricellular proteins to the greatest extent (Below et al., 2022). The new system may provide some hints for whether a more accurate ECM for endometrial organoid and stromal cells growth and expansion can be established. HGF, FGF10 secreted by surrounding stroma (Sugawara et al., 1997; Chen et al., 2000; Chung et al., 2015). Decidual cells also responding to hormone signaling secrete decidual prolactin (PRL) and C-X-C motif chemokine ligand 14 (CXCL14) (Rawlings et al., 2021). Coculture model is a more suitable method to investigate the relationship between pre-implantaion embryo and receptive endometrium.

## Application in Gynecological Disease and Endometrial Receptivity

Patient-derived endometrial organoids maintaining genomic landscape form donors have also been widely used in drug testing and *in vivo* transplantation. Compared with the primary and cell line cultures, the establishment of pathological models have many advantages such as similar tumorous marker expression and mutational landscape which can guide individual therapy (Drost et al., 2017). From the perspective of morphology, normal endometrial organoids usually form circular spheroid a hollow lumen while the patient-derived organoids form a compacted and solid one. Ectopic organoids derived from endometriosis (ECT-O) grow more slowly than those organoids from health, usually it takes 7–14 days on account of harder dissociation conditions. ECT-O recapitulates endometriosis phenotype and disease-related traits. It is surprising that organoids derived from endometrial carcinoma have low formation efficiency and limited expansion compared with organoids come from precancerous lesions with high formation efficiency and can be passaged steadily for more than 6 months (Boretto et al., 2019). Evidences show that histological, molecular features, invasive and metastatic capacity are similar with primary origins (Kiyohara et al., 2016; Kopper et al., 2019). It has been demonstrated in organoids which are established from ovarian cancer, drug testing can be conducted on established organoids derived from ovarian cancer indicates the individual drug response (Maenhoudt et al., 2020). Consistently, Witte et al. revealed that organoids from ovarian cancer genetically resembled the primary tumorous and drug testing reflected patients’ clinic response (de Witte et al., 2020). Organoids treated with oestrogen, progesterone and cyclic adenosine monophosphate (cAMP) can accurately mimic implantation window and help investigate the paracrine communication of the embryo secretome in maternal-fetal interface. Glycodelin A (GdA) which considered an endometrial receptive marker, was significantly upregulated in receptive endometrial epithelial organoids after stimulating with embryo-conditioned culture medium (Focarelli et al., 2018; Luddi et al., 2021). Coincide with changed receptive marker and ultrastructure of epithelial glandular organoids treated with medium conditioned using the human embryo, the biochemical and clinical pregnancy rate of patients undergoing IVF significantly elevated when receiving the embryos in self-spent medium compared with those receiving the embryos with fresh medium (Wang et al., 2021). Mimicking the process that how human blastocyst interacts with endometrium, endometrial organoids can be seeded in 2D to form an open-faced endometrial layer (OFEL). Exposure to oestrogen and progestogen, 2D OFEL expressed the receptive marker and can attached to the stem cell derived blastoid, while the nonreceptive OFEL cannot. (Boretto et al., 2017; Wanxin Wang et al., 2020). As happening *in vivo*, blastocyst didn’t attach to nonreceptive OFEL (Kagawa et al., 2022).

## Conclusion

The endometrial organoid model is of significance for mimicking physiological and pathological conditions. Based on the crosstalk occurred between the epithelium and stroma during not only the normal menstrual cycle but also in embryo implantation, a coculture model has been promoted, and a new type of artificial synthesized ECM has been developed based on *in vivo* feature.

Endometrial organoids usually possess similar histologic origin characteristics. Normal endometrial tissue and decidual tissue are usually used for the physiological characteristics, and tumor tissue possesses a strong proliferation ability. Organoid culture involves many signaling pathways, which may influence the size and number of organoids. With the development of single-cell sequencing, some signaling has been shown to impact stem/progenitor cell self-renewal and determine the fate of differentiating cells. The problem is that normally effective genome editing methods cannot reach ideal efficiency with the solid ECM. Thus, most gene knock-in and knockout experiments depend on the remove of ECM and the dispersion of spheroids into single cells. However, gene editing *in situ* with Matrigel/Hydrogel intact might be the best choice. Based on ethical restrictions, endometrial organoids and descendant models may be the best alternative, and understanding the intrinsic structure and mechanism would benefit the clinical practice.
