# Supplementary material for: Human Endometrial Organoids: Recent Research Progress and Potential Applications
Source: Front Cell Dev Biol. 2022 Feb 15;10:844623. doi: 10.3389/fcell.2022.844623 (PMC8885623; doi:10.3389/fcell.2022.844623)
Supplement: Supplementary file 1 [file Table1.DOCX]

| Dekkers 2017 | Final Concentration | Boretto 2017 | Final Concentration | Rawlings 2021 | Final Concentration |
| --- | --- | --- | --- | --- | --- |
| Advanced DMEM/ F12 | 1X | DMEM/ F12 | 1X | Advanced DMEM/ F12 | 1X |
| N2 supplement | 1X | N2 supplement | 1X | N2 | 1X |
| B27 supplement minus vitamin A | 1X | B27 supplement minus vitamin A | 1X | B27 | 1X |
| Primocin | 100 μg/ml | PS | 1X | Antibiotic -Antimycotic | 1X |
| L- glutamine | 2 mM | Glutamax | 2 mM | L- glutamine | 2 mM |
| A83- 01 | 500 nM | A83- 01 | 500 nM | A83- 01 | 500 nM |
| Recombinant human EGF | 50 ng/ml | Recombinant human EGF | 50 ng/ml | EGF | 50 ng/ml |
| Recombinant human Noggin | 100 ng/ml | Recombinant human Noggin | 100 ng/ml | Noggin | 100 ng/ml |
| Recombinant human Rspondin- 1 | 500 ng/ml | Recombinant human Rspondin- 1 | 200 ng/ml | R- spondin- 1 | 500 ng/ml |
| Recombinant human FGF- 10 | 100 ng/ml | Recombinant human FGF- 10 | 50 ng/ml | FGF- 10 | 100 ng/ml |
| Recombinant human HGF | 50 ng/ml | ITS | 1X | HGF | 50 ng/ml |
| Nicotinamide | 10 nM | Nicotinamide | 1 mM | Nicotinamide | 10 mM |
| N- acetyl- L- cysteine | 1.25 mM | N- acetyl- L- cysteine | 1.25 mM | N- acetyl- L- cysteine | 1.25 mM |
|  |  | E2 | 2 nM |  |  |
|  |  | p38 inhibitor SB202190 | 10 μM |  |  |
|  |  | WNT3A | 200 ng/ml |  |  |
|  |  | Y27632 | 9 μM |  |  |

Supplementary Table 1：Culture media composition
